# Obstetrics

**Published:** 1860-05

**Authors:** 


### OBSTETRICS.

Cephalic Version of the Fœtus in the Uterus by External Version.—It may be known to some of our readers that Dr. Ignatius Langer, a practitioner of Davenport, was expelled, in 1859, by the Scott County Medical Society of Iowa, on account of alleged unprofessional conduct—one of the principal charges against him being for an attempt to rectify malposition of the fœtal head by external manipulation. Without pretending to know anything whatever of the merits of the controversy, or case in question, and without any desire to side with either party, we cannot refrain from publishing Dr. Langer's citation of authorities in justification of his practice. The subject of cephalic version is just now attracting much attention, in consequence of the proceedings of the Scott County Medical Society, and it is proper, therefore, that we should give our readers the benefit of an outline of the opinions of some of the obstetrical writers in regard to it. It is seldom that there is a public controversy from which, ultimately, good does not come.

"From my limited library, I can produce the reports of a number of successful cases, and more than twenty medical authors of older and modern schools, who wrote on the subject; also the favorable opinion of quite a number of living professors in medical schools of good standing, who are considered all over the educated world as masters in the art. I name them alphabetically, for who will presume to arrange such masters in any other manner?

"BUSH, late Professor at the University of Berlin, Principal Director of the Lying-in Institution, etc.—Text-book on Obstetrics, third edition, Berlin, 1836, p. 400, § 894–96; an extensive treatise on version by manipulation.

"BUSH AND MOSER.—Bush and Moser's Hand-book on Obstetrics, Berlin, 1843, vol. iv. pp. 509–513. On turning by external manipulations, aided by placing the pregnant woman in a proper situation.

"BRAITHWAITE'S RETROSPECT, 1857, p. 214, part 35, July, 1857.

"CAZEAUX, P. Prof. in the Faculty of Medicine in Paris; President of the Medical Society.—A Theoretical and Practical Treatise on Midwifery, translated from the fifth French edition, by Dr. Bullock, Philadelphia, 1859, pp. 772–75. Version by the head has been advised: first, before the labor; second, during labor, and prior to the rupture of the membranes, etc. On the whole, therefore, we believe that the cephalic version may and ought to be attempted. Third, in presentation of the trunk whether before the labor, or during the labor, and before the rupture of the membranes, etc.

"CHIARA, Braun a Spath, Prof. in Vienna, Austria.—In Prof. Scanzoni's Contribution to Obstetrics, Wurtzburg, 1854, p. 319. In the lying-in institutions of Vienna, in 1852, seven thousand eight hundred and thirty-five mothers had seventy cross presentations. Spontaneous versions during the last three months of pregnancy, nineteen times; during the commencement of parturition, twice observed; and the cephalic version by external manipulations, ten times performed.

"CHURCHILL, Fr. Professor of Midwifery in Dublin, one of the Pres. of the Obstetric Society.—Theory and Practice of Midwifery, third London edition, 1855, p. 300, § 508–12–18, on cephalic version.

"CANNSTADT.—A yearly report on the progress of medicine in general. Editors and Professors, Dr. Sherer Wishchoos, Dr. Eisenthann. Wurzburg, 1853, vol. iv. p. 427.

"CONTRIBUTORS TO OBSTETRICS.—New Series: Berlin, 1850; vol. xxviii. p. 259. Two cases of cephalic version, by external manipulation alone; both children alive. Thirteen cases by external and internal manipulations, eleven alive; turning on the feet seventy-five, out of them forty-one alive.

"DETROIT.—Carsus on Midwifery, according to the plan of Dr. C. S. Weiss, Magdeburg and Berlin, 1844, vol. i. p. 331, § 55.

"ELLIOT, GEORGE T.—College of Physicians and Surgeons, New York. Obstetric Clinic, October 14th, 1859. This talented observer, who has taken great pains to inform himself, at the schools of both hemispheres, speaks thus: 'On cross-birth, Mattei, a Corsican Professor of Obstetrics, has proposed an intelligent method of procedure in this class of cases, which is based on sound reasoning, and upon the well-recognized phenomena of reflex action, displayed by the fœtus in utero. Its practice demands an accurate knowledge of the various positions of the fœtus in utero, and a habit of appreciating its various parts, through the abdominal walls; two circumstances which will not allow its benefits to be of service in the hands of men not accustomed to the practice of obstetrics, but which do not invalidate the value of the principle. I have been in the habit of teaching this principle of Mattei's for three years, and it is about time for it to creep into the text-books.'—(Vide *Medical Press*, vol. ii. No. 18.)

"ESTERLE, Professor of Obstetrics in the University of Trient.—In Schmidt's Yearly Reports, vols. civ. and x. p. 76, 1859. In an extensive article this writer insists on the high value of the cephalic version by external manipulations as preventative to premature labor, in cases originating from cross-birth.

"LUMPE, Emeritus Assistant of the First Clinic and Lying-in Hospital.—Cursus on Practical Midwifery, with special regard to the doctrines of the Obstetrical School of Vienna, 1843. With regard to Cephalic Version by External Manipulations, p. 75, § 57. His opinion is so exalted that he considers its happy performance as a sign of the highest obstetric education and skill.

"LONDON LANCET, (American edition,) November 29th, 1856, p. 585.—Martin, late Professor of Jena, now of Berlin, as Prof. Churchill of Dublin states: a very interesting memoir has been published by Prof. Martin, of Jena, on turning by external manipulation alone, founded on thirty-four cases, seven of which occurred to himself.

"MEIGS, Professor of Midwifery in Jefferson Medical College.—Obstetrics, the Science and Art of; third edition; Philadelphia, 1856, pp. 436 and 514.

"MICHAELIS.—The Narrow Pelvis, by Litzman, Prof. of Midwifery and Director of the Lying-in Institute at Kiel. Leipzig, 1851, p. 180, § 212.

"NÆGELE, Prof. of Obstetrics in the University of Heidelberg.—Pathologies, Therapie of Birth, part second, p. 337, § 644; Mainz, 1849. The treatment recommended by Wigand, which seeks to effect the changing of the malposition of the fœtus, into the longitudinal position simply by means of external manipulations and a suitable placing of the pregnant woman, has received more general approbation than the version on the head by means of internal manipulations. Sometimes we can make use of this method already in the last stage of pregnancy, and during parturition it can be applied up to the wane of the second period. It ought, however, never to remain untried, as soon as the malposition of the fœtus is ascertained, at the beginning of the parturition, because its effects are more mild than the version by means of internal applications of the hand; and because, also, where it does not effect the purpose, it does not in any way interfere with any other proceeding which it may be necessary to adopt afterwards.

"NŒGGERATH AND JACOBI'S valuable Contributions to Midwifery and Diseases of Women and Children, with Reports on the Progress of Obstetrics, etc., New York, 1859, p. 124. Dr. Hallett's Report, p. 293. The Report of Dr. Meisner, the obstetric veteran of Saxony, Prof. in Leipzig. Cephalic version is preferred by some practitioners, as giving the child the best chance; but it can only be tried when hastening the delivery is not an object. The author resorted to it in six cases. In four of the three hundred and fifty-one cases, turning was performed by external manipulations.

"Nœggerath.—The Operation of Turning by External Manipulations, considered from a Historical and Practical Point of View; with Cases. The *New York Journal of Medicine*, No. 99, November, 1859, contains (pages 329–347) a very interesting article which is very creditable to the author, who closes as follows: 'We feel it our duty to call upon the profession to remember, that there is such a thing as turning by external manipulation, and that those who have practiced it most often were men, the names of whom we only pronounce with a feeling of deepest admiration.'

"Osiander, Professor of Obstetrics and Director of the Lying-in Institute of Gottingen.—Elements of Midwifery, as a manual to his lectures: Gottingen, 1802; vol. ii. p. 35.

"Ramsbotham, Obstetric Physician to the London Hospital, Lecturer on Obstetrics.—By Wm. V. Keating: Philadelphia, 1859; p. 360. Thus Wigand has stated: 'That it is possible, before the waters have escaped, to change the position of the head or even the presentation by external manipulation alone, founded on thirty-four cases, seven of which occurred to himself.'

"Rosshirt, Professor and Director of the Lying-in Institute, Erlangen.—The Obstetrical Operations: Erlangen, 1842. Version by External Manipulations, p. 141, § 82.

"Scanzoni.—Text-book of Obstetrics, Preparatory Operations, chapter 6, Cephalic Version (a) by External Manipulations.

"Siebold, Professor of Obstetrics, Director of the Lying-in Institute at Gottingen.—Explanation to the Obstetrical Plates of Theory and Practice of Obstetrics. Second edition: Berlin, 1842; p. 212. To have recourse to external manipulations as soon as the malposition of the fœtus, through the abdominal walls, is ascertained.

"Ibidem, Siebold, Corresponding Member to the Batavian Society for Science and Art.—In answer to a question on Midwifery in Japan, by one of his former pupils, Mimazunza, now practical physician at Nangasaki, translated in Siebold's Journal, vol. vi. art. 3, p. 687.

"Smith, Wm. Tyler.—A Course of Lectures on Obstetrics, delivered at St. Mary's Hospital, London, with Practical Annotations, by Aug. K. Gardener. Second edition: New York, 1858; p. 674. Cephalic version is very much aided by external manipulation, particularly when the uterus and abdominal walls are sufficiently thin to allow of the different parts of the fœtus being readily felt. Cases are even recorded by Martin, of Jena, and others, in which rectification and alteration of malpresentation have been effected by external manipulation alone.

"Velpeau.—An Elementary Treatise on Midwifery, etc. On Cephalic Version by External Manipulations. 'I had already followed this precept before I was acquainted with Professor Wigand's doctrine,' etc., etc.

"Wigand, in his Essay, Three Cases of Version by External Manipulations, addressed to the Medical Faculties of Paris and Berlin. Hamburg, 1812; p. 35. Wigand's Description of the Birth of Men; Berlin, 1820."

